# A Service Based Adaptive U-Learning System Using UX

**DOI:** 10.1155/2014/109435

**Published:** 2014-07-23

**Authors:** Hwa-Young Jeong, Gangman Yi

**Affiliations:** ^1^Humanitas College, Kyung Hee University, 1 Hoegi-dong, Dongdaemun-gu, Seoul 130-701, Republic of Korea; ^2^Department of Computer Science & Engineering, Gangneung-Wonju National University, Gangwon-do 220-711, Republic of Korea

## Abstract

In recent years, traditional development techniques for e-learning systems have been changing to become more convenient and efficient. One new technology in the development of application systems includes both cloud and ubiquitous computing. Cloud computing can support learning system processes by using services while ubiquitous computing can provide system operation and management via a high performance technical process and network. In the cloud computing environment, a learning service application can provide a business module or process to the user via the internet. This research focuses on providing the learning material and processes of courses by learning units using the services in a ubiquitous computing environment. And we also investigate functions that support users' tailored materials according to their learning style. That is, we analyzed the user's data and their characteristics in accordance with their user experience. We subsequently applied the learning process to fit on their learning performance and preferences. Finally, we demonstrate how the proposed system outperforms learning effects to learners better than existing techniques.

## 1. Introduction

E-learning can provide learning materials to users without quickly and conveniently. For users, e-learning is the process of studying through learning materials contained in digital media resources, and these media include the internet, computers, satellite broadcasts, videos, interactive TV, CDs and so on [1]. Also, it represents an efficient ideal approach to flexible and cost-effective development for the learning process since it can be used without any limitation related to distance and time of usage [2]. Furthermore, the development of e-learning provides new possibilities to improve learning performance and is leading to dramatic changes in the education area. These changes affect not only the educational institutions, but also the enterprises themselves [3]. Actually, within an e-learning system, in addition to the evaluation of technology itself, it is important factor for learning system developer and faculty to design and evaluate the learning process and materials for providing an effective learning service as well [4, 5]. In light of this consideration, many learning researchers have studied methods to apply new possibilities within various computer environments and networks [4]. U-learning (ubiquitous learning) was introduced by many researchers. P. Brusilovsky et al,.[6] and Joiner et al. [7] proposed a way to explore the design of situated online educational experiences—in particular to investigate the effect of having a goal on motivation in the ubiquitous computing environment. Tsai et al. [8, 9] show a way to display students' qualitatively which represents a different conception of U-learning. Through the proposed method, they wanted to show that the findings can assist researchers in the learning area to develop more efficient and adaptive U-learning system services. They also propose U-learning environments for enhancing students' learning performance and to encourage learners to use better approaches to their learning.

On the other hand, there is a lot of research focusing on how to improve a user's learning performance when he or she studies the course or learning units via the learning system. In order to provide a user-oriented learning process to users (students) for this purpose, educational researchers proposed an adaptive learning process. Adaptive learning is one of these efficient learning models. It needs to store and maintain information on learners when the developers of a learning system construct the user model. Making a user model implies gathering users' information and transferring it into the model. In an adaptive learning area, one of the students' features frequently used for adaptation purposes can depend on their learning style. In recognition of the fact that individuals study in different ways, a body of research and techniques has been developed, which attempts to identify individual variations while satisfying different learning preferences and style [10].

The techniques for system development have been changing in the recent years. A service-based system as an efficient kind of service technology has been introduced. The services are becoming the standard for software development and the deployment of a service oriented computing (SOC) based paradigm. Along with their associated technologies, SOAP: a message exchange protocol for service interactions, WSDL (web services description language): a language for describing service interfaces, and UDDI: a repository for dynamic service application and publication are all services which provide a powerful process for integrating existing software applications on the web, programming language, execution platform, or transport protocol [11].

In this research, we propose a service-based adaptive learning system using UX (user experience). To consider UX, we identify a user's characteristics and analyze her/his learning pattern, preferences, and learning performance as a learning style. All the processes for learning courses were stored in the learning database in the service provider's server included LMS (learning management system) and LCMS (learning contents management system). And this system provides the function that the system developer can just access the business process and construct them when he/she implements the learning system. Using this system, the user or learner is able to use the learning system via the internet using smart phones at any time.

## 2. Related Works

### 2.1. Ubiquitous Computing

**Figure 1:**
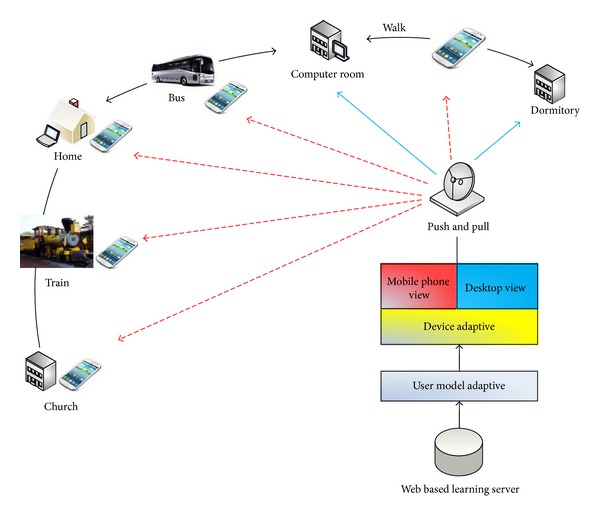
The conceptual design for a ubiquitous learning system by Chen et al. [12].

Over the past few years, many researchers and developers have been working on system development and management in ubiquitous computing environments. A service is a programmatically available application process available via the internet [13]. The main factor of ubiquitous computing is to create and develop user centric services and an application orientated computing environment. Actually, these environments are different from the existing computing models since a space within the environments supported by related hardware and software promotes interactive information exchange between learners, and the space [14] is connected to the service applications. Hence, research in this area is most often related to application physical domains or specific applications. Mark Weiser's original work in ubiquitous computing can be categorized as driven by technology and network communication. The systems were developed and built around a vision of available technologies [15]. For the learning system in ubiquitous computing, Chen et al. [12] proposed the scenario and conceptual design of a ubiquitous learning system as shown in Figure 1. Their model includes adaptive devices and user model components. Furthermore, they attempted to show the scenario whereby users can learn using desktop PCs, laptops, handheld PCs, and cellular phones in the ubiquitous learning environment.

### 2.2. Service Based Application

A service is software application logic which is available over the internet [16]. Services, and more in general service oriented architectures (SOAs), are an emerging technology and architecture of choice for implementing distributed computing systems and performing system application integration. The basic principles of SOAs consist of modularizing system process logics and their functions and exposing them as a service that are specified using program languages and interoperate by the standard protocols [17]. Services are self-contained system software modules and components that expose specific functionality over the internet, such that other modules and applications can use them by means of established internet protocols and data formats such as HTTP and XML [18].

**Figure 2:**
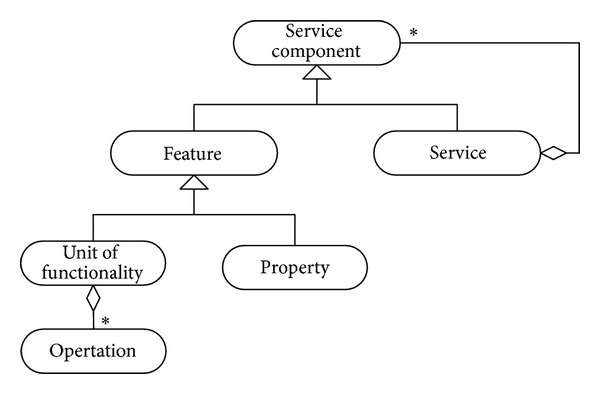
The relationship between services and their features.

Weiss et al. [19] depicted the structure of the relationship between services and their features as shown in Figure 2. In their diagram, each feature is a module of a closely related system or program operations that can be invoked through the service. The services can be constructed from lower-level services which combine to result in each service.

To describe the interfaces of services, WSDL is used but does not provide versioning. Therefore it can be challenging for versioning to evolve a service interface through its life cycle. Usually, it requires managing an instance of a service for each version, separately. This increases the number of service instances, making them difficult to manage and control. To manage and control the services, a UDDI (universal description, discovery, and integration) registry is used [20]. UDDI means a standardized model for service registry. Also, it is the information model, and the service supports API for registering and publishing services when the service consumers request API for services to UDDI [21].

### 2.3. User Experience (UX)

Users' characteristics are regarded as one the most important factors to be considered in the software and other product industries. Recently, the importance of user experience (UX) such as a combination of users' sensibilities, emotions, and affections has been emphasized [22]. User Experience (UX) can be defined as a result of the presentation, system performance, interactive behavior, functionality, assistive capabilities of an interactive application and system, and both of hardware and of software. Also, it is a result of, or information pertaining to the user's prior experiences, attitudes, characteristics, skills, tastes, habits, and personality [23]. That is, human perception on UX originates can come from evolution of the user's internal states which is affective states and cognitive processes. It affects human interactions with choice decision making [24]. Also, UX can be affected by a user's emotional state, such as happiness, disgust, surprise and love, and any other feeling [25].

### 2.4. Adaptive Learning System

Web-based instruction (WBI) researchers have considered a flexible learning curriculum sequencing control to support adaptable, variable, personalized learning programs [26]. Basically, E-learning is a web-based system that makes learning information or knowledge available to learners without time restrictions or physical locations. Generally, traditional teaching resources are textbooks. Typically, it guides the learners to follow fixed sequences on the contents to other subject related to the current one during the learning.

Online learning has given more advantages than traditional education, that is, to support flexible learning process, various learning materials, efficient and immediate evaluation ways for learning performance, including feedback to the learner. However, online learning should be concerned with how to provide learning materials and units to the learner adaptively and personally. Further considerations should include learning time and materials involved in running e-Learning environments [27].

In this context, what is adaptive learning? Adaptive learning is defined as a capability to change learning processes or materials according to learner's study ability or preference. Therefore, the adaptive learning environment includes the individual in that she/he has personal characteristics which make her/him unique. This learning is considered to be an alternative to learner/user oriented learning process or user tailored learning. Also, teachers or learning system developers have encouraged the development of teaching and learning units and processes towards a dynamic learning process. Adaptive learning systems are to feature learner preferences, interests, learning materials, and browsing behaviors to provide personalized learning services [28–30].

## 3. Service Based Adaptive Learning System

This research aims to provide a learning process according to user's learning ability via a web service on the internet. To account for a user's learning ability and their learning characteristics, learning style was used.

In general, learning styles has been considered as being the way people prefer to learn. This group of individual characteristics is close to cognitive and preference style, but more narrow in scope because of its focus on a learner's learning [6, 7]. A learner's intrinsic motivation, goal setting and resultant class attendance can all be considered important factors related to an individual's learning style [8, 9]. Brusilovsky and Millán [6, 7] proposed user models considering learning style as being divided into three layers: what is being modeled, how this information is structured, and how different kinds of models are maintained.

**Figure 3:**
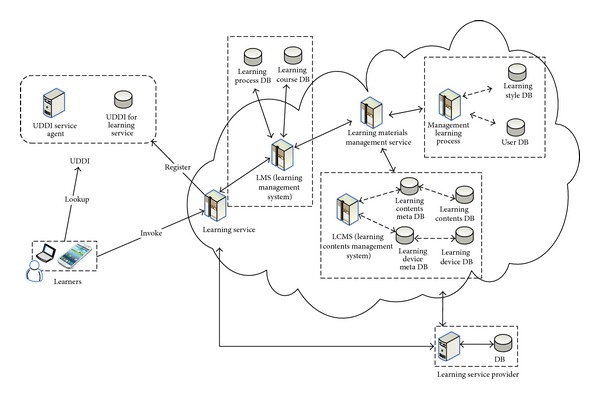
Service based learning materials management and learning process architecture.

**Figure 4:**
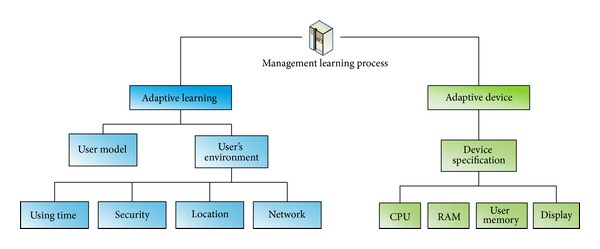
The structure of the* management learning process*.

In this research, we consider learning style in order to improve a learner's learning performance.  Figure 3 shows a structure for the proposed U-learning system. This architecture consists of 5 servers for the services:* learning service, LMS (learning management system), LCMS (learning contents management system), learning materials management service, and management learning process*. The core modules are* management learning process, Learning materials management service, and Learning service*. All the learning materials provide the service by learning service provider. The learning style is located in* learning style DB* with* management learning process*. In the* management learning process*, we use a UX based* User model* in order to provide an adaptive learning service. This process consists of 2 modules,* Adaptive learning* and* Adaptive device*, as shown in Figure 4. Adaptive learning deals with the process which considers user model and her/his use environment such as* using time, security, location, *and* network*. The* Adaptive device* is for the user's current device environment. The* Device specification *can be information for the user's instrument such as smart phone, mobile phone, lab top, and hand-held PC. The Internet environment is the network status concerning how fast the user's internet is.

**Figure 5:**
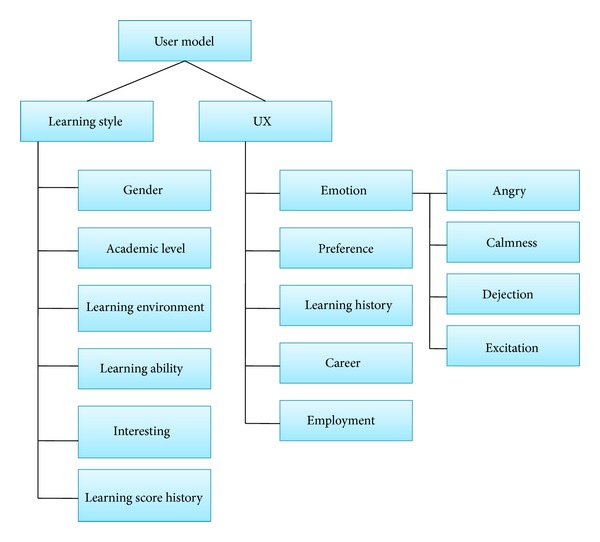
Service based learning materials management and learning process architecture.

Figure 5 shows the detailed structure for the User model in Figure 4. The* User model* consists of 2 main factors,* Learning style* and* UX*.* Learning style* related to the general status of users such as* gender, academic level, learning environment, learning ability, interests and learning score history*. On the other hand, UX is about the user's sensual environment and related to factors such as* emotion, preference, learning history, career, and employment*.* Emotions* can include* anger, calmness, dejection, and excitation *and the* preference* refers to what does user enjoy learning.

**Figure 6:**
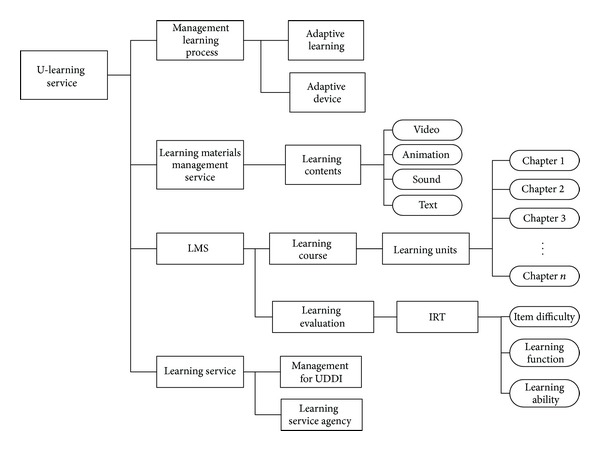
The service management structure for learning style, materials, course, process, and history.

Finally, we can summarize the proposed system, an adaptive U-learning system using UX, as being like the one depicted in Figure 6. In this structure, the service management has mainly 4 processes: a* management learning process, a learning materials management service, LMS, and a learning service*. In particular,* management learning process *is for adaptive learning and includes a user model and UX.

*A Learning materials management service* handles the control and management of many kinds of learning materials such as video, animation, sound, and text.* LMS* includes 2 modules, a learning course and evaluation. We use* IRT (item response theory)* for* learning evaluation* to analyze user's learning ability from learning score and its effect.* Learning service* deals with the process for UDDI and handing the learning service provided to the user.

## 4. Experimental Results

In order to conduct the current research, we used two groups, a control group for the existence process and an experimental group for the proposed process. The participants consisted of 180 students who are studying in Kyunghee University, Seoul, Korea. All of them use smart phones and use these to access their learning units for their study. All participants took a pretest for the same learning course. We divided participants into two groups (the control and the experimental) with similar pretest score distribution according to the pretest results. For evaluating and analyzing the test, we use SPSS statistical software. The results of independent samples *t*-tests are provided in Table 1.

Table 2 shows the mean score and standard deviation between two groups is similar.

Table 2 shows the result of Levene's Test that the null hypothesis is satisfied under the significant level of 0.05 and *F* = 11.636 < 0.002; that is, the variance between the two groups is equal. Furthermore, there is no difference between two groups under the significant level of 0.05 and *P* = 0.213 > 0.05.

For the experimental group, we provide UX based learning style to them. Otherwise, for the control group, they completed the learning progress following the existing learning process without any learning style and UX. Paired sample *t*-test results between the pre- and posttest of the control group are shown in Table 3.  Table 4 displays that there is no significant difference under the significant level of 0.05 and *P* = 0.582 > 0.05.

Table 5 displays the paired sample *t*-test results for the experimental group. The mean score of the posttest increased about 14.6 points.  Table 6 shows that the difference in mean scores is significant at the significant level of 0.05 and *P* = 0.000 < 0.05. Therefore, we are able to conclude that the proposed learning system is more effective than the existing one.

Table 7 shows the result of the independent samples *t*-test for the posttest.

Consequently, Table 7 shows the mean score for the experimental group is higher than the control group by about 15.8 points. The result of Levene's test (see Table 8) shows that the null hypothesis is rejected under the significant level of 0.05 and *F* = 1.584 > 0.219. It means that the variance between the two groups is different. Moreover, there is a significant difference between two groups at the significance level of 0.05 and *P* = 0.000 < 0.05. Therefore, we can conclude that the learning performance using the proposed system (experimental group) is more efficient than that of the existing system.

## 5. Conclusions

In this paper, we proposed a service based adaptive U-learning system. This system consists of 5 servers for the services:* learning service, LMS, LCMS, learning materials management service, and management learning process*. The main modules are a* management learning process, a learning materials management service, and a Learning service*. The* management learning process* has a process for adaptive learning. The* Learning materials management service* handles learning materials from LCMS, such as video, animation, sound, and text. Finally, we considered the* learning service* process for the UDDI and handing the learning service to the user.

In order to provide an adaptive learning process, we used learning style and UX. The* learning style DB* with* management learning process* stored learning style in a user model that has 2 main factors,* learning style* and* UX. *learning style means user's characteristics such as* gender, academic level, learning environment, learning ability, interest, and learning score history*. And UX means user's sensual environment and includes factors such as* emotion, preference, learning history, career and employment*.

To get the experimental result, we grouped participants into two groups, a control group using the existing system and an experimental group using the proposed system. The experimental results showed the proposed system is more efficient than the current system.

Further investigation is necessary to ascertain the method or system necessary to provide learning content suitable for learner's abilities and consider detailed factors in UX such as emotion or feeling. This is because the learner's emotional state during the learning process can have an influence on learning performance.

## Figures and Tables

**Table 1:** Group statistics of pretest results in the control group and experimental group.

Group	*N*	Mean	Std. deviation	Std. error mean
Control	90	66.9333	3.59497	.92822
Experimental	90	68.2667	1.86956	.48272

**Table 2:** Independent samples *t*-test of pretest results between two groups.

	Levene's Test for equality of variance		*t*-test for equality of means						
	*F*	Sig.	*T*	df	Sig. (2-tailed)	Mean difference	Std. error difference
								Lower	Upper
Equal variance assumed	11.636	.002	−1.274	28	.213	−1.3333	1.04623	−3.47644	.80978
Equal variance not assumed			−1.274	21.056	.216	−1.3333	1.04623	−3.50874	.84207

**Table 3:** Paired sample statistics for the control group between the pre-and posttest.

	Mean	*N*	Std. deviation	Std. error mean
Pre-test results	66.9333	90	3.59497	.92822
Post-test results	67.0667	90	3.86313	.99746

**Table 4:** Paired sample *t*-test for the control group between the pre- and posttest.

	Paired differences							
	Mean	Std. deviation	Std. error mean	95% Confidence interval of the mean		*t*	dt	Sig. (2-tailed)
				Lower	Upper			
Pre—post	−.13333	.91548	.23637	−.64031	.37364	−.564	14	.582

**Table 5:** Paired sample statistics for the experimental group between the pre- and post-test.

	Mean	*N*	Std. Deviation	Std. Error Mean
Pretest results	68.2667	90	1.86956	.48272
Posttest results	82.8667	90	2.97289	.76760

**Table 6:** Paired sample *t*-test for the experimental group between the pre- and posttest.

	Paired differences							
	Mean	Std. deviation	Std. error mean	95% Confidence interval of the mean		*t*	dt	Sig. (2-tailed)
				Lower	Upper			
Pre—post	−14.60000	2.26148	.58391	−15.85237	−13.34763	−25.004	14	.000

**Table 7:** Group statistics of posttest results for two groups.

Group	*N*	Mean	Std. deviation	Std. error mean
Control	90	67.0667	3.86313	.99746
Experimental	90	82.8667	2.97289	.76760

**Table 8:** Independent samples *t*-test of posttest results for two groups.

	Levene's test for equality of variance		*t*-test for equality of means						
	*F*	Sig.	*t*	df	Sig. (2-tailed)	Mean difference	Std. Error Difference
								Lower	Upper
Equal variance assumed	1.584	.219	−12.553	28.000	.000	−15.80000	1.25862	−18.37817	−13.22183
Equal variance not assumed			−12.553	26.276	.000	−15.80000	1.25862	−18.38581	−13.21419
